# Heart failure disease management programs in Austria 2019: A systematic survey of the Heart Failure Working Group and the Working Group for Cardiological Assistance and Care Personnel of the Austrian Society of Cardiology

**DOI:** 10.1007/s00508-020-01615-y

**Published:** 2020-02-18

**Authors:** Gerhard Poelzl, Bettina Fetz, Johann Altenberger, Margret Fritsch, Johann Auer, Elfriede Stachl, Armin Boehmer, Heinrich Frauendorfer, Christian Ebner, Friedrich Geyrhofer, Stefan Pötz, Annette Prim, Hannes Alber, Renate de Grandis, Christiane Drack, Martin Hülsmann, Heart Failure Working Group and the Working Group for Cardiological Assistance and Care Personnel of the Austrian Society for Cardiology

**Affiliations:** 1 Department of Internal Medicine III, Cardiology & Angiology, Medical University of Innsbruck, Anichstraße 35, 6020 Innsbruck, Tyrol Austria; 2 Landesinstitut für Integrierte Versorgung – LIV, Innsbruck, Tyrol Austria; 3 Rehabilitation Center Grossgmain, Salzburg, Austria; 4 University Clinic of Internal Medicine II, Paracelsus Medical University, Salzburg, Austria; 5 Department of Internal Medicine, St. Joseph’s Hospital Braunau, Braunau, Upper Austria Austria; 6 Department of Internal Medicine 1, Krems University Clinic, Krems, Lower Austria Austria; 7 Department of Internal Medicine 2, Hospital of the Order of St. Elizabeth, Linz, Upper Austria Austria; 8 Department of Internal Medicine, Hospital Hochsteiermark, Bruck/Mur, Styria Austria; 9 Department of Internal Medicine & Cardiology, Klinikum Klagenfurt am Wörthersee, Klagenfurt, Carinthia Austria; 10 Rehabilitation Center Bad Ischl, Bad Ischl, Upper Austria Austria; 11 University Clinic of Internal Medicine II, Medical University of Vienna, Vienna, Austria

**Keywords:** Heart failure, Disease management plan, Multidisciplinary management

## Abstract

Heart failure (HF) is common and is associated with high morbidity, mortality and high health expenditure. A multidisciplinary disease management plan (DMP) can reduce morbidity and mortality, save costs and improve the quality of life. In Austria, three HF-specific DMPs are currently in a project phase and four established DMPs are active. Although programs are widely heterogeneous with respect to their intervention type, they pursue the same interventional goal by supporting seamless care between inpatient and community care settings with a multidisciplinary team. This survey presents a systematic survey of the HF-specific DMPs in Austria. Disparities between programs are highlighted and discussed. The nationwide establishment of HF-specific DMPs that integrate primary care and cardiology services including a regulation of the remuneration of stakeholders and program infrastructure is needed to decrease the burden of HF for both the individual and society.

## Introduction

Heart failure (HF) is a common chronic medical problem that is associated with considerable morbidity and mortality [1–3]. The prevalence of HF continuously rises due to changing demographics and better survival from cardiovascular diseases earlier in life. Meanwhile, there is abundant evidence to demonstrate the efficacy of a multidisciplinary team approach to meet the increasing complexity of HF management [3–7].

In 2017 the Heart Failure Working Group and the Working Group for Cardiological Assistance and Care Personnel of the Austrian Society of Cardiology published a position paper on the need for disease management programs (DMP) specific for heart failure in Austria [8]. That article defined essential elements of a DMP that were essentially based on recommendations made by the Heart Failure Association of the European Society of Cardiology (ESC) [3, 9]. The publication attracted much attention from healthcare providers and political representatives and enhanced an ongoing public debate about optimal management of chronic heart failure in Austria. Nevertheless, a comprehensive concept for nationwide coverage with heart failure-specific DMPs is still pending.

This article presents a systematic survey of currently active heart failure-specific DMPs in Austria. To ensure a high degree of clarity in presenting the heterogeneity of individual programs and allow for comparison, the summary is essentially based on the aforementioned position paper of the Austrian Society of Cardiology [8] and on a system of classification developed by The American Heart Association’s Disease Management Taxonomy Writing Group [10]. This publication consciously refrains from presenting efficacy and cost effectiveness analyses since final or interim evaluation is not yet available for all programs. Final assessment of the programs will be addressed in a future publication.

Currently, seven individual DMPs are operating in six regions in Austria ranging from Carinthia (Klagenfurt) to Lower Austria (Krems), Salzburg, Styria, Tyrol, and Upper Austria (Braunau, Linz) (in alphabetical order). Kardiomobil Kärnten (January 2019–December 2020), Integrierte Versorgung von Patienten mit Herzinsuffizienz in Oberösterreich – IVH OÖ-Pilot (January 2017 to December 2019), and KardioStabil Braunau (June 2017 to unlimited) are still in a project phase, while Kardiomobil Salzburg (since 2008) [11], the Krems model (since 2008) [12] and HerzMobil Tirol (2012–2017 in a project phase, established since July 2017) [13–19] and HerzMobil Stmk (since January 2019) are already established programs.

A summary of heart failure-specific DMPs in Austria including the number of included patients as of May 2019, the proportion of women, and the drop-out rate are given in Table 1–4. Various aspects and disparities between programs are highlighted and discussed in detail. HerzMobil Tirol and HerzMobil Stmk as well as Kardiomobil Salzburg and Kardiomobil Kärnten are largely identical with a few exceptions and thus are mostly discussed as units.

**Table 1:** Taxonomy of heart failure-specific DMPs in Austria – part 1

	KardioMobil Kärnten	Krems Model	Kardiomobil Salzburg	HerzMobil Stmk	HerzMobil Tirol	KardioStabil Braunau	IVH-OÖ-Pilot
Model and form of intervention	Home visiting program	Hybrid model	Home visiting program	Hybrid model	Hybrid model	Hybrid model	Home and office-based
	Nurse-based multidisciplinary	Clinic based and telehealth care multidisciplinary	Nurse-based multidisciplinary	Home visiting and telehealth care and multidisciplinary network	Home visiting and telehealth care and multidisciplinary network	Clinic based and telehealth care	Multidisciplinary
	Transitional care intervention (from acute to primary care)	Transitional care intervention (from acute to primary care)	Transitional care intervention (from acute to primary care)	Transitional care intervention (from acute to primary care)	Transitional care intervention (from acute to primary care)	Multidisciplinary	Transitional care intervention (from acute to primary care)
						Transitional care intervention (from acute to primary care)	
Status	Project-limited	Program	Program	Program	Program	Project-limited	Project-limited
Patient population	Hospitalization for AHF	HFrEF pts. primarily following hospitalization for AHF	Hospitalization for AHF	Hospitalization for AHF	Hospitalization for AHF	Hospitalization for AHF	Hospitalization for AHF
			Non-hospitalized pts with AdHF	Non-hospitalized pts with AdHF and NTproBNP >1500 pg/ml	Non-hospitalized pts with AdHF and NTproBNP >1500 pg/ml	Non-hospitalized pts with AdHF	AdHF pts. hospitalized for AHF within the last 6 months
			NYHA II and non-adherence a/o difficulties w treatment optimization				
Exclusion criteria	Dementia	Dementia	Pts. refractory to therapy	Life expectancy <6 months	Life expectancy <6 months	Life expectancy <6 months	Pts. refractory to therapy
	Non-mobile pts	Non-mobile pts	Nursing home residents	Multimorbidity (Charlson Index >6)	Multimorbidity (Charlson Index >6)	Dementia	Nursing home residents
	Multi-morbidity	Lack of willingness to participate	Dementia	Dementia	Dementia	Lack of willingness to participate	Dementia
	Lack of willingness to participate		Lack of willingness to participate	Lack of willingness to participate	Lack of willingness to participate		Lack of willingness to participate
Intervention content	Patient education	Patient education	Patient education	Patient and family education	Patient and family education	Patient education	Patient education
	Monitoring of disease symptoms	Monitoring of disease symptoms via telephone contacts	Focus on pt. self-management (patient diary, self-management, i.e. with diuretics)	Development of individual patient care plans	Development of individual patient care plans	Monitoring of disease symptoms via telephone contacts	Monitoring of disease symptoms
	Optimization of treatment based on prevailing guidelines in cooperation with primary care physician	Optimization of treatment based on prevailing guidelines	Optimization of treatment based on prevailing guidelines	Optimization of treatment based on prevailing guidelines	Optimization of treatment based on prevailing guidelines	Optimization of treatment based on prevailing guidelines	Optimization of treatment based on prevailing guidelines
				Home telemonitoring of disease symptoms, body weight, blood pressure, heart rate, and subjective well-being	Home telemonitoring of disease symptoms, body weight, blood pressure, heart rate, and subjective well-being		
				Medication reminder system	Medication reminder system		
Environment	Home-based outpatient	Home-based, outpatient	Home-based, outpatient	Home-based, outpatient	Home-based, outpatient	Home-based, outpatient	Home-based, outpatient
Number of included patients by May 2019	19	Approx. 2200, 180 in parallel	Approx. 2000	53	350 600 pts/yr. with completion	75	197
Female (%), drop-out rate (%)	33.8/7.7	34/9	39/5	19/3	33/5	34/5	31/3

*DMP* disease management program, *AHF* acute heart failure, *HFrEF* heart failure with reduced ejection fractioin, *NYHA* New York Heart functional class, *AdHF* advanced heart failure, *NTproBNP* N-terminal pro-B-type natriuretic peptide, *HF* heart failure, *QoL* quality of life, *KCCQ* Kansas City Cardiomyopathy Questionnaire, *EHFBScB-9* 9-item European Heart Failure Self-care Behaviour Scale, *ECG* electrocardiogram, *NÖGUS* Niederösterreichischer Gesundheits- und Sozialfonds, *SGKK* Österreichische Gesundheitskasse – Kundenservice Salzburg UMIT, Private University for Health Science, Medical Informatics and Technology, Tyrol, *OÖGKK* Österreichische Gesundheitskasse – Mein Gesundheitszentrum für physikalische Medizin und Rehabilitation Linz, *ELGA* Electronic Health Records Austria, *KAGES* Steiermärkische Krankenanstaltengesellschaft m.b.H., *KABEG* Kärntner Landeskrankenanstalten-Betriebsgesellschaft

## Model and form of intervention (Table 1)

Most programs use hybrid tactics that combine either home visiting (HerzMobil Tirol/Stmk) or clinic-based (Krems Model, KardioStabil Braunau) approaches and telehealth care. Telehealth care includes regular telephone contacts with patients (Krems Model, KardioStabil Braunau) or mobile telephone-based home telemonitoring systems (HerzMobil Tirol/Stmk) [13]. HerzMobil Tirol also implements a broad network of resident physicians for home-based care delivery. Kardiomobil Salzburg/Kärnten is designed primarily as a nurse-based home visiting program and the IVH OÖ-Pilot is conceptionally a home and office-based program.

It is the primary goal of all programs to prevent readmissions among patients transitioning from hospitalization for acute heart failure (AHF) to outpatient healthcare facilities and to improve the quality of life (QoL). The transition is considered the most vulnerable period with the highest rates of readmission and mortality. In this sense all programs are designed as transitional care interventions.

## Patient population (Table 1)

Most programs include patients who are hospitalized for AHF irrespective of the ejection fraction and the underlying cause and who are now transitioning to community care. The Krems Model restricts inclusion to patients with reduced ejection fraction. Additionally, outpatients with advanced HF (AdHF) are included in all programs except Kardiomobil Kärnten. Patients with serious or life-threatening comorbidities, dementia, need for permanent nursing home care, advanced frailty or communication problems, and lack of willingness to participate in a program are excluded. HerzMobil Tirol also explicitly excludes patients with a high degree of comorbid conditions (Charlson index >6) since experience in the early phase demonstrated that the program was unable to cope with this complexity.

## Intervention content (Table 1)

Comprehensive patient education, monitoring of disease symptoms and optimization of treatment based on established HF guidelines are common to all programs.

A specialty-trained HF nurse meets with the patient while still in hospital (Krems Model, HerzMobil Tirol/Stmk, KardioStabil Braunau, IVH OÖ-Pilot) or at the latest within 2 weeks after discharge (Kardiomobil Salzburg/Kärnten) for extensive patient education including weight monitoring, medication adherence, symptom recognition, and diet. Patient education addresses patients either individually or as a group. In HerzMobil Tirol/Stmk patient education is supported by a patient brochure and also includes training in the handling of a provided mobile telephone, weight scale and blood pressure monitor. In both programs the nurse meets again with the patient within 1 week after discharge to repeat the training and ensure the availability of prescribed medication. The mobile telephone displays a constantly updated medication reminder on a daily basis over the course of the entire program.

In all programs an individual patient care plan is provided at hospital discharge and transmitted to the primary care physician. Individual treatment goals that include self-care behavior, medication adherence, QoL, physical activity, decongestion status and HF readmissions are set between the patients and the providers.

In the Krems Model and KardioStabil Braunau, patients’ symptoms are monitored via office visits at the heart failure outpatient clinic and resident network physicians, respectively, in addition to regular telephone contacts for both programs. The INH OÖ-Pilot also provides regular office visits with resident network physicians and patient support from the local mobile support/social service.

In Kardiomobil Salzburg/Kärnten the HF nurse meets with the patient at home within 1‑2 weeks after discharge at the discretion of the referring hospital or resident physician. At home visits special attention is paid to patient training involving family members and registration of the medication. Visits are conducted according to the Kardiomobil protocol and an electronic report is created and made available to the resident physician instantly via DaMe (A1, Vienna, Austria), the electronic health platform used state-wide.

In HerzMobil Tirol the patient is assigned to a resident network physician and regular office visits are scheduled. In HerzMobil Tirol/Stmk physiologically relevant patient-associated parameters including heart rate, blood pressure, weight, well-being, and adherence to medication are obtained daily from patients via a remote electronic monitoring system.

## Delivery personnel (Table 2)

**Table 2:** Taxonomy of heart failure-specific DMPs in Austria – part 2

	KardioMobil Kärnten	Krems Model	Kardiomobil Salzburg	HerzMobil Stmk	HerzMobil Tirol	HerzMobil Tirol	IVH-OÖ-Pilot
Delivery personnel	Hospital-based cardiologists and specialists for internal medicine Specialty-trained HF nurses interacting with resident physician	Hospital-based cardiologists and specialists for internal medicine Specialty-trained HF nurses Program coordinator	Hospital-based cardiologists and specialists for internal medicine Specialty-trained HF nurses Networks of resident physicians: specialists for internal medicine and general practitioners Program coordinator	Hospital-based cardiologists and specialists for internal medicine Specialty-trained HF nurses Program coordinator	Hospital-based cardiologists and specialists for internal medicine Specialty-trained HF nurses Networks of resident physicians: specialists for internal medicine and general practitioners Program coordinator	Hospital-based cardiologists and specialists for internal medicine Specialty-trained HF nurses Networks of resident physicians: specialists for internal medicine and general practitioners Program coordinator	Hospital-based cardiologists and specialists for internal medicine Specialty-trained HF nurses Networks of resident physicians: specialists for internal medicine and general practitioners Local mobile support/social service
Means of communication	*Patient to caregiver:* face to face telephone	*Patient to caregiver:* face to face telephone	*Patient to caregiver:* face to face	*Patient to caregiver:* face to face telephone internet	*Patient to caregiver:* face to face telephone internet	*Patient to caregiver:* face to face telephone	*Patient to caregiver:* face to face
	*Caregiver to caregiver:* face to face telephone internet	*Caregiver to caregiver:* face to face (nurse-hospital-based cardiologist) telephone physician letter (nurse/cardiologist general practitioner)	*Caregiver to caregiver*: telephone face to face	*Caregiver to caregiver:* face to face telephone internet	*Caregiver to caregiver:* face to face telephone internet	*Caregiver to caregiver:* telephone face to face	*Caregiver to caregiver:* telephone face to face
Intensity and complexity	6 months *Frequency of contact:* Nurse meets patient within 1 week after discharge Follow-up visits are individually scheduled telephone contacts when needed	6 months *Frequency of contact:* Every 4 weeks at the heart failure outpatient clinic Alternating every 4 weeks telephone-based nurse contact	3 (6) months *Frequency of contact:* 1–3 nurse visits In selected cases (at the discretion of the HF specialist) 3 more visits and prolongation of project possible	–	3 (6) months *Frequency of contact:* At a minimum, nurse meets patient before discharge from hospital, at week 1 and week 12 Network resident physician meets patie nt at weeks 1, 4 and 12 Telemedical transmissions daily Telephone and internet contacts when needed	3–6 months *Frequency of contact:* At a minimun, nurse meets patient before discharge from hospital Telephone FU at week 1–2 Telephone contacts when needed	12 months *Frequency of contact:* Network physician meets patient at least once per quarter Final visit at the heart failure outpatient clinic at the end of the program Nurse meets patient according to the network physicians’ advice
Definition of the process and exact role of caregivers	Yes	Yes	Yes	Yes	Yes	Yes	Yes

### Nurses

All programs are based on multidisciplinary teams that integrate specialty-trained nursing staffs. Nurses graduate from a comprehensive heart failure educational course at AZW Innsbruck (Training Center West for Allied Health Professions) that is certified by the ESC Heart Failure Association and the Austrian Society of Cardiology. Their duties include patient education and outpatient evaluation to relay clinically relevant findings to the patient’s physician, thereby effecting more intensive management of the disease.

In the Krems Model and KardioStabil Braunau nursing staff are located in the hospital and patient contacts are made by telephone. In Kardiomobil Salzburg/Kärnten and IVH OÖ-Pilot nurses personally meet with patients at home at various intervals according to the patients’ needs. In IVH OÖ-Pilot nurses are supported by members of the local mobile support and/or social services, who underwent specific heart failure training.

In HerzMobil Tirol/Stmk nurses supervise equipment-obtained and remotely transmitted physiologic data plus information on subjective well-being and medication intake on a daily basis. Supervision is facilitated by an automatic event detection program that signals the need for therapeutic decisions triggered by missing values and off-limit measurements, where limits are individually defined. Alerts that cannot be handled by the nurse are forwarded to the network physician [13]. Also, nurses personally meet with patients at home within the first week after discharge for retraining in the presence of relatives and to check the availability of the prescribed medication. Another home visit is planned after 3 months for the purpose of assessing negotiated treatment goals. In the meantime, contacts via telephone and/or encrypted message service (EMS) and optional unplanned home visits are provided as needed.

### Physicians

Hospital-based cardiologists or specialists for internal medicine are involved in all programs. It is their duty to include patients and design individualized treatment plans that are immediately transferred to all stakeholders. In the Krems Model and HerzMobil Stmk the patient is followed in an HF outpatient clinic on a regular basis. In HerzMobil Tirol/Stmk individual limits of physiologic data are also provided for the analyzing filter of the web-based HerzMobil telehealth software until necessary changes are made by the resident network physician. In all programs any serious decisions on patient management that become necessary during the program period are the responsibility of hospital-based cardiologists in the respective heart failure outpatient clinic and/or the medical program director. The IVH OÖ-Pilot provides a mandatory office visit in a heart failure outpatient clinic 1 year after inclusion and optional interim visits, if needed.

In KardioStabil Braunau, IVH OÖ-Pilot and HerzMobil Tirol a network of resident specialists for internal medicine and/or general practitioners is actively involved in patient management with particular attention focusing on the gradual optimization of HF medication. Network physicians are advised to follow a management plan delivered at discharge from hospital.

In Kardiomobil Salzburg/Kärnten the patient’s resident physician (general practitioner or internist/cardiologist) is the primary contact person for the heart failure nurse. Contacts mostly address optimization of heart failure medication and laboratory controls. Nurses report on disease trajectory and relevant notes in the patient’s medical diary. Hospital-based cardiologists and the medical program director are available to the resident physician and the nurse when complex problems arise. In Kardiomobil Kärnten regular office visits in the heart failure outpatient clinic are planned after 6 and 12 months.

In HerzMobil Tirol an initial consultation with the resident network physician is scheduled within the first week after discharge and two more visits at weeks 4 and 12. Consultations include laboratory analysis with special attention to renal function and electrolytes. It is the responsibility of the network physicians to optimize HF treatment based on established HF guidelines and to react in a timely manner in the case of disease deterioration and thus prevent rehospitalization. Network physicians review the summarized transmitted data from assigned patients on a weekly basis or immediately in the case of upgraded alerts. Necessary therapy adjustments according to predefined algorithms are transferred to patients either by telephone, the nurse or as part of an additional office visit to ascertain timely interventions [13].

### Coordinator

A medical coordinator orchestrates all stakeholders and manages efficient cooperation of the involved partners. This is a full-time position in HerzMobil Tirol held by a heart failure nurse and a part-time position staffed by either a nurse or a hospital-based physician in all other programs.

Kardiomobil Salzburg is coordinated by a non-medical staff member of the organizational platform AVOS.

## Means of communication (Table 2)

Augmented face to face interaction is the means of communication between care providers and patients in all programs. Telephone contact for the purpose of monitoring clinical symptoms, reinforcing educational content, offering encouragement, and responding to patient questions is implemented in all programs except Kardiomobil Kärnten and IVH OÖ-Pilot. HerzMobil Tirol/Stmk also uses a remote electronic monitoring system (HerzMobil telehealth system) that works with near field communication (NFC)-enabled mobile telephones and Bluetooth low energy (BLE)-enabled medical devices to record measurements of patients’ weight, blood pressure, heart rate, and information on subjective well-being and medication intake. Encrypted message service (EMS) via mobile telephone is also used to exchange messages between patients and caregivers [14, 15].

In all programs communication among caregivers, which is essential for interaction between program elements, is conducted face to face and via telephone. In HerzMobil Tirol/Stmk information is also shared instantly on the web-based HerzMobil telehealth system.

In Kardiomobil Salzburg information is stored on the web-based Kardiomobil software and shared with resident physicians via DaMe.

## Intensity and complexity (Table 2)

### Duration

Duration of the provided structured intervention ranges from 3 (KardioStabil Braunau, HerzMobil Tirol/Stmk, Kardiomobil Salzburg) to 6 (Kardiomobil Kärnten, Krems Model) or 12 (IVH OÖ-Pilot) months. KardioStabil Braunau, HerzMobil Tirol/Stmk and Kardiomobil Salzburg allow a one-time extension to 6 months if negotiated treatment goals are not achieved after 3 months.

### Frequency/periodicity

In HerzMobil Tirol/Stmk three regular face-to-face meetings with the nurse (one in-hospital, one home visit within 1 week after discharge and another one at the end of the program) and three regular office meetings with the network physician are scheduled. Event-related telephone/EMS contacts are intended. If necessary, additional home or office visits are possible.

In KardioMobil Salzburg regular home visits with the nurse are planned for 1–3 dates within 3 months. Another 1–3 visits are possible at the discretion of the resident physician or the medical program director. In IVH OÖ-Pilot an office visit with the resident network physician is intended at least once per quarter year. Home visits by the nurse are necessary in about 15% of included patients. In the Krems Model an office visit in the heart failure outpatient clinic is scheduled every month, alternating with phone contacts made by the nurse.

### Complexity

All programs offer a mix of various individual components resulting in intermediate to high complexity. Individualized discharge planning and extensive self-management education are common to all programs.

The Krems Model and KardioStabil Braunau involve one hospital each. Patient education is provided still during hospitalization and disease symptoms are monitored mostly by telephone contacts. Optimization and adjustment of medication is managed at prescheduled visits at the heart failure outpatient clinic (Krems Model) or at prescheduled office visits with the resident network physician (KardioStabil Braunau).

KardioMobil Salzburg includes eight hospital and two cardiac rehabilitation centers throughout the state. Patient education and monitoring of disease symptoms are managed by a mobile nurse during home visits. Treatment optimization is stipulated in the individual patient care plan and executed by the resident physician. The heart failure nurse monitors the work off the care plan and the patient’s adherence to the medication plan. This is a key element in the electronic protocol and is reported to the resident physician. Disease monitoring is based on patient self-monitoring (blood pressure, heart rate, weight) and is recorded in a medical diary. The system implements six nurses, who are assigned to dedicated geographic regions and who collaborate with the regional hospitals. Each nurse is in regular contact with the regional hospital-based cardiologists/specialists for internal medicine and also offers in-hospital education, if requested. Organization and execution of nurse visits as well as data management are processed via the Kardiomobil software system.

The IVH OÖ-Pilot includes four hospitals. Patient education is managed by a cardiologist and a HF nurse while patients are still in hospital. Monitoring of symptoms and constant advising of patients are handled by the local support and social service. Treatment optimization is handled by the resident network physician or the heart failure outpatient clinic.

HerzMobil Tirol/Stmk currently involve seven and two hospitals, respectively. Patient education is provided during hospitalization and during home visits. Disease monitoring is managed via telemonitoring, where telemedical data are reviewed daily by a nurse and weekly by a resident network physician (HerzMobil Tirol) and a hospital physician (HerzMobil Stmk). The complexity of these programs arises from a network of HF nurses and resident network physicians, who communicate via an internet-based platform (HerzMobil telehealth system) operated in the local eHealth infrastructure of Tirol/Stmk. Resident network physicians define and implement treatment optimization at prescheduled office visits or else by timely reacting to alerts provided by the telemonitoring system.

## Monitoring of program outcomes/outcome measures (Table 3)

**Table 3:** Taxonomy of heart failure-specific DMPs in Austria – part 3

	KardioMobil Kärnten	Krems Model	Kardiomobil Salzburg	HerzMobil Stmk	HerzMobil Tirol	KardioStabil Braunau	IVH-OÖ-Pilot
Clinical outcomes	QoL, NYHA class and medication at study entry, at last visit and 12 months (KCCQ, EHFBScB-9) Hospitalization and mortality at month 12 Echo at baseline, at last visit and after 12 months Assessment of goal attainment at first and last week	QoL, Echo, NT-proBNP, medication, and labs at study entry and at 6 month assessment of negotiated treatment goals by HF nurse, network physician and patients	Hospitalization and mortality	QoL, echo and medication at study entry, at 3 months NT-proBNP, renal function and electrolytes at study entry, at 4 and 12weeks Assessment of negotiated treatment goals by HF nurse, network physician and patients	QoL and medication at study entry, at 3 months and 12 NT-proBNP, renal function and electrolytes at study entry, at 4 and 12 weeks Hospitalization and mortality at 3 and 12 months Assessment of negotiated treatment goals by HF nurse, network physician and patients	Hospitalization and mortality at 12 months Assessment of negotiated treatment goals by HF nurse, network physician and patients	QoL at study entry and at 12 months Hospitalization and mortality at months 12 NT-proBNP, echo, ECG, KCCQ, and pt. adherence at 12 months Final assessment by the HF outpatient clinic at month 12
Quality control	Structured introduction to the program for physicians and nurses Involvement of specialty trained HF nurses Regular advanced training for involved physicians	Structured introduction to the program for physicians and nurses Regular advanced training for involved physicians and nurses Regular case conferences External quality control (NÖGUS)	Joure fix for HF nurses and team leader, 2 ×/year Joure fix for HF nurses, network physicians and team leader, 2 ×/year Advanced training for involved physicians and nurses Involvement of specialty trained HF nurses Performance report to SGKK, 1–2 ×/year	Structured introduction to the program for physicians and nurses Involvement of specialty trained HF nurses Regular quality circles for involved physicians (2–3 ×/year)	Structured introduction to the program for physicians and nurses Involvement of specialty trained HF nurses Regular quality circles for stakeholders (4 ×/year) Regular advanced training for involved physicians and nurses Monthly case conferences (HF nurses) External quality control (UMIT) 1 ×/year	Regular quality circles for stakeholders 2 ×/year Involvement of specialty trained HF nurses Regular advanced training for involved physicians	Structured introduction to the program for physicians and nurses Involvement of specialty trained HF nurses Regular quality circles for stakeholders
Algorithm for troubleshooting	24/7 telephone hotline handled by hospital cardiologists	Telephone hotline handled by heart failure nurses	*Nurses have to contact physicians at different levels:* Resident physician Kardiomobil—physician in each hospital	Automatic alerts to HF nurse and network physician in case limit values of transferred data are exceeded or explicit pt. request Predefined reaction in terms of pt. counseling and optimization of HF medication	Automatic alerts to HF nurse and network physician in case limit values of transferred data are exceeded or explicit pt. request Predefined reaction in terms of pt. counseling and optimization of HF medication	HF nurse informs hospital-based network physician in case limit values are exceeded	24/7 telephone hotline handled by hospital cardiologists
Transition to regular care after the end of the program	Final report Written report at every visit Continuation of pt. diary recommended	Final report Ongoing telephone contact 3 months after end of the program	Electronic report in the Kardiomobil software written by the heart failure nurse Individual agreement between network physician, HF nurse and patient	Final report by the network physician	Final report by the network physician Scale and blood pressure monitor are provided free to patients Ongoing data transfer and handling in terms of pt. diary are possible	Transfer of pts. to regular care	Final report Continuation of pt. diary recommended

Monitoring of outcomes is an integral element of all programs. Assessment of negotiated treatment goals is common to all programs. Hospital readmissions and mortality at the end of the program and at 12 months are registered in most but not all programs. Some programs also control for QoL, laboratories including NTproBNP, and echocardiography at various time points. Details are shown in Table 1. Additionally, quality of HF medication and adherence to medication are recorded in HerzMobil Tirol/Stmk [17] and KardioMobil Kärnten.

## Quality control (Table 3)

Quality assurance (QA) and quality control (QC) are integral to all programs, although with some inconsistencies. Common to all programs is the QA warranty by providing an exact definition of the respective process flow and the roles of involved caregivers, the majority of which are specialty-trained. All programs offer regular advanced retraining for physicians and nurses. An introductory workshop is mandatory for first-time stakeholders and regular quality circles and case conferences are provided in most programs. Quality circles are used to implement necessary optimization steps and restructuring of the respective program. QC is based on benchmark evaluation, where assessed parameters differ between programs (Table 1). Heart failure rehospitalizations and mortality are assessed in all but two programs (Krems Model, HerzMobil Stmk.) and can be used for future cost-effectiveness analysis. Optimization of heart failure therapy and patient adherence to medication is controlled for in HerzMobil Tirol and KardioMobil Kärnten. Kardiomobil Salzburg is required to submit an annual performance report to the funding institution. Two programs are subjected to an annual external quality control by the provider (Krems Model) and the Private University for Health Science, Medical Informatics and Technology, Tyrol (HerzMobil Tirol) [19].

## Algorithm for troubleshooting (Table 3)

Timely reaction to clinical deterioration of involved patients is ensured differently. In all programs the nurse who monitors disease symptoms by phone or face-to-face contacts, alerts the network physician, if needed. In Kardiomobil Salzburg the patient’s resident physician is informed first. In the case of serious problems the alert is upgraded to the hospital-based network physician. In KardioStabil Braunau the hospital-based network physician is the primary contact person, IVH OÖ-Pilot and Kardiomobil Kärnten provide a 24/7 telephone hotline that is handled by a hospital-based cardiologist.

In HerzMobil Tirol/Stmk the nurse responds to an automatic event detection program that signals the need for therapeutic actions based on patient notes and/or missing or off-limit measurements, where limits are individually defined by the network physician. Alerts that cannot be handled by the nurse are upgraded to the network physician. In both programs predefined algorithms propose the reaction in cases of patient counselling and adjustment of HF medication.

## Transition to regular care (Table 3)

Transition of patients from DMP to regular community care at the end of the program is supported by a patient report. The Krems Model offers ongoing telephone contact with the nurse for an additional 3 months. HerzMobil Tirol gives the patients a free scale, blood pressure monitor and mobile telephone and offers ongoing data transfer and handling in terms of a patient diary.

## Provider and incentives (Table 4)

**Table 4:** Taxonomy of heart failure-specific DMPs in Austria – part 4

	KardioMobil Kärnten	Krems Model	Kardiomobil Salzburg	HerzMobil Stmk	HerzMobil Tirol	KardioStabil Braunau	IVH-OÖ-Pilot
Provider	KABEG commissioned and authorized by Gesundheitsfonds of Carinthia	NÖGUS, Universitätsklinikum Krems	Arbeitskreis für Vorsorgemedizin – AVOS	KAGES commissioned and authorized by Gesundheitsfonds Steiermark	Institut für Integrierte Versorgung Tirol, Tirol Kliniken commissioned and authorized by Tiroler Gesundheitsfonds	KH St. Josef Braunau	OÖGKK
Initiation	January 2019	Pilot project started in 2006, program established in 2008	Program established in 2008	Pilot project started in April 2017, program established in January 2019	Pilot project started in 2012, program established in July 2017	October 2017	January 2017
Incentives	Project funding (2 years) by Gesundheitsfonds of Carinthia	Program funding (20 physician h/week and 30 nurse h/week) by NÖGUS	Program funding by Salzburg State and insurances carriers 70:30	Established flat-rate remuneration of network physicians per pt—StGKK and all Austrian nationwide insurance companies Full-time employment of HF nurses (State of Styria/insurance companies 50:50) Cost recovery of the program infrastructure (State of Styria)	Established flat-rate remuneration of network physicians per pt—TGKK and all Austrian nation-wide insurance companies Full-time employment of HF nurses (State of Styria/insurance companies 50:50) Cost recovery of the program infrastructure	Project funding by the local hospital operator	Project funding by State of Upper Austria/OÖGKK 50:50
Future directions	Project evaluation in 2021 Establishment of the DMP Expansion of inclusion of outpatients and hospitalized patients from referral hospitals	Maintenance of established quality	Integration of a telemedical component Electronic connection to all hospitals Establishment of networks of resident physicians and advanced training Definition of an exact program duration	Stepwise inclusion of all regions in Styria Establishment of networks of resident physicians Establishment of an overall direction of the DMP Connection of HerzMobil Stmk. with ELGA Connection of HerzMobil Stmk. with Medocs (medical information system)	Stepwise inclusion of all regions in Tyrol Current status: 5 of 9 regions included (completion expected by the end of 2021) Implementation of a cardiac rehabilitation program Connection of HerzMobil Tirol with ELGA Extension of HerzMobil to a comprehensive cardio-vascular disease management program (hypertension, CHD, peripheral arterial disease) Extentension of HerzMobil to residential care home for the elderly	Integration of the project into a multi-regional program operated by the State of Upper Austria and OÖGKK	Project evaluation in 2020 establishment of the DMP
Further information	Kardiologie.klagenfurt@kabeg.at	www.kup.at/kup/pdf/9582.pdf armin.boehmer@krems.lknoe.at	www.kardiomobil.at	www.patienten-portal.kages.at/cms/ziel/7760191/DE/	www.liv-tirol	Johann.auer@khbr.at	https://vertragspartner.ooegkk.at/herzinsuffizienz

*DMP* disease management program, *AHF* acute heart failure, *HFrEF* heart failure with reduced ejection fractioin, *NYHA* New York Heart functional class, *AdHF* advanced heart failure, *NTproBNP* N-terminal pro-B-type natriuretic peptide, *HF* heart failure, *QoL* quality of life, *KCCQ* Kansas City Cardiomyopathy Questionnaire, *EHFBScB-9* 9-item European Heart Failure Self-care Behaviour Scale, *ECG* electrocardiogram, *NÖGUS* Niederösterreichischer Gesundheits- und Sozialfonds, *SGKK* Österreichische Gesundheitskasse – Kundenservice Salzburg UMIT, Private University for Health Science, Medical Informatics and Technology, Tyrol, *OÖGKK* Österreichische Gesundheitskasse – Mein Gesundheitszentrum für physikalische Medizin und Rehabilitation Linz, *ELGA* Electronic Health Records Austria, *KAGES* Steiermärkische Krankenanstaltengesellschaft m.b.H., *KABEG* Kärntner Landeskrankenanstalten-Betriebsgesellschaft

KardioStabil Braunau and the Krems Model are provided by the local hospital operator (KH St. Josef Braunau, Niederösterreichischer Gesundheits- u. Sozialfonds – NÖGUS). Kardiomobil Kärnten, IVH OÖ-Pilot and HerzMobil Stmk are provided by the local hospital operator (Kärntner Krankenanstalten-Betriebsgesellschaft – KABEG, Oö Gesundheitsholding GmbH – GESPAG/Ordensklinikum Linz GmbH/JKU Linz, Steiermärkische Krankenanstaltgesellschaft – KAGes) commissioned and authorized by the healthcare fund of the respective state. In HerzMobil Tirol, operational matters are outsourced by the healthcare fund of the State of Tyrol to Landesinstitut für integrierte Versorgung – LIV. Kardiomobil Salzburg is outsourced and operated by Arbeitskreis für Vorsorgemedizin – AVOS.

Temporary limited project funding for KardioStabil Braunau is provided by the hospital operator, for Kardiomobil Kärnten and IVH OÖ-Pilot by the Health Care Fund of the particular state. Temporary unlimited funding for the Krems Model is warranted by the hospital operator and for Kardiomobil Salzburg by the Health Care Fund of the State of Salzburg and the health insurance carriers of the state of Salzburg. In HerzMobil Tirol/Stmk the program infrastructure is provided by the Health Care Fund of the particular state. Full-time employment of nurses is funded by a 50:50 agreement between the Health Care Fund of the particular state and the compulsory health insurance carriers. Network physicians are remunerated at a flat rate per patient that is paid by the compulsory health insurance carriers.

## Future directions (Table 4)

All programs that are still in a project phase are pursuing final evaluation and definite establishment of the program. Kardiomobil Salzburg is aspiring to implement a telemedical component and set up a defined network of resident physicians. HerzMobil Tirol/Stmk are striving to integrate all hospitals in the respective state and to connect the program to the national electronic health record—ELGA. HerzMobil Tirol is in the process of testing the implementation of a cardiac rehabilitation element into the program.

## Conclusion

Prevalence and cost of care for HF will continue to increase because of the aging of the population. Efficacy has been demonstrated for a multidisciplinary team approach to meet the increasing complexity of HF management [3, 4, 6, 7, 9].

In Austria seven HF-specific DMPs are currently active. Programs are widely heterogeneous with respect to intervention type. This is mostly due to the geographic and political diversity and to differences in the local health infrastructure between Austria’s states. Also, geographical conditions in terms of rural-urban distribution and also budgetary requirements substantially impact the respective intervention type. Still, they pursue the same intervention goal by supporting seamless care between inpatient and community care settings in a multidisciplinary team. Programs are either hospital-based or home-based and consistently integrate specialty-trained nursing staff. Depending on the mix of individual components, complexity ranges from regular telephone contacts and/or home visits to mobile telephone-based remote telemonitoring systems involving networks of resident physicians. While three programs are still in a project phase, four programs are already well established. In two programs the remuneration of stakeholders is clearly set down in contracts with compulsory health insurance companies.

Inclusion criteria are defined with some blurs in all programs. Patients with multimorbidities and in the end-of-life period are currently not included in any of the programs. Care for these patients exceeds the capacity and financial restrictions of programs in this early phase but is a worthwhile goal for the future. Quality assurance is guaranteed in all programs and measures for quality control are implemented.

Finally, it needs to be noted that despite the fact that it is very gratifying that in Austria some DMPs for heart failure are already running, this is still only a first step in the right direction. The ultimate goal must be the nationwide establishment of HF-specific DMPs that integrate primary care with cardiology services including clear-cut regulation of the remuneration of stakeholders and program infrastructure. This is the most likely way to diminish the burden of HF for both the individual and society. It is our hope that presentation of already existing heart failure-specific DMPs will stimulate and encourage the stepwise institution of similar facilities in all Austrian states in order to meet local needs and requirements.
